# Size Distributions of Microplastics in the St Louis Estuary and Western Lake Superior

**DOI:** 10.1021/acs.est.3c10776

**Published:** 2024-05-02

**Authors:** Ariyah Thomas, Joseph Marchand, Guenter D. Schwoerer, Elizabeth C. Minor, Melissa A. Maurer-Jones

**Affiliations:** † Department of Chemistry and Biochemistry, University of Minnesota Duluth, 1038 University Dr. , Duluth , Minnesota 55812, United States; ‡ Large Lakes Observatory and Department of Chemistry and Biochemistry, University of Minnesota Duluth, 2205 East Fifth St. , Duluth , Minnesota 55812, United States

**Keywords:** microplastics, size, morphology, polymer composition, microFTIR, power law, Lake Superior, freshwater

## Abstract

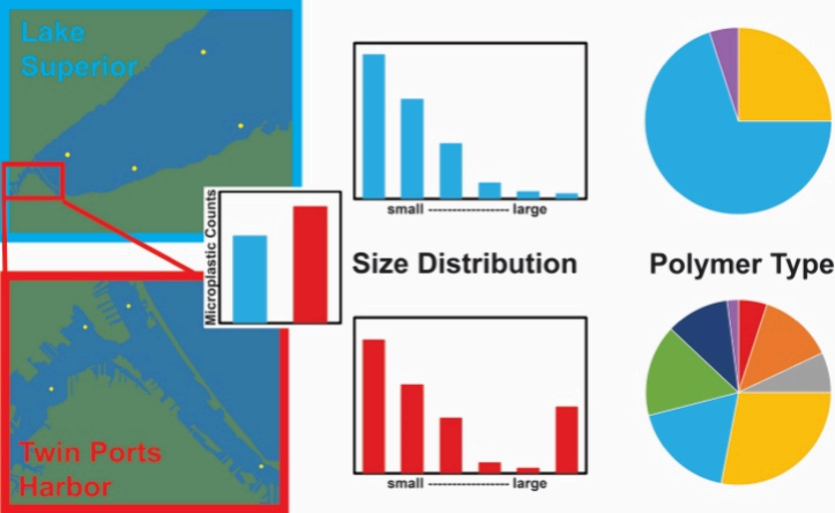

Identifying the sources and fate of microplastics in natural systems has garnered a great deal of attention because of their implications for ecosystem health. This work characterizes the size fraction, morphology, color, and polymer composition of microplastics in western Lake Superior and its adjacent harbor sampled in August and September 2021. The results reveal that the overall microplastic counts are similar, with the harbor stations ranging from 0.62 to 3.32 microplastics per liter and the lake stations ranged from 0.83 to 1.4 microplastics per liter. However, meaningful differences between the sample locations can be seen in the size fraction trends and polymer composition. Namely, the harbor samples had relatively larger amounts of the largest size fraction and more diversity of polymer types, which can be attributed to the urbanized activity and shorter water residence time. Power law size distribution modeling reveals deviations that help in the understanding of potential sources and removal mechanisms, although it significantly underpredicts microplastic counts for smaller-sized particles (5–45 μm), as determined by comparison with concurrently collected microplastic samples enumerated by Nile Red staining and flow cytometry.

## Abstract 2

Microplastic size distribution and polymer composition indicate different sources and processing with lake vs harbor location.

## Introduction

In 2019, 460 million tonnes of plastic were commercially produced, much of this satisfying the global need for inexpensive, simple to produce, and single-use products. While plastic products serve a myriad of purposes and their production has been projected to grow exponentially, the extensive use of plastic has inadvertently created plastic waste, with 55% of the amount of plastic produced in 2019 being mismanaged or deposited into landfills in 2019.^1^ The primary source of plastic contamination in the environment is improper disposal of plastic, polluting water sources with bulk debris, microplastics, and nanoplastics.^2^

Plastics can cause serious biological and physical effects on aquatic organisms and humans, and these effects are often dependent on plastic particle size. Some of the ecological effects caused by plastics, including microplastics, on aquatic life include intestinal blockage, tissue implantation, stimulated growth, and transporting of other pollutants.^3^ The effects of microplastics on these organisms depend in part upon the plastics being of ingestible size and upon whether they can translocate into tissues of the organism in question. Smaller microplastics and nanoplastics thus appear to have a wider array of effects on biota.^4^ For example, Fringer et al. showed that nanopolystyrene (159 nm diameter) caused a dose-dependent effect on the amount of riboflavin secreted from an ecologically relevant bacterial model.^5^ In another study, polystyrene nanoparticles were shown to impact metabolites made by the microbiome in zebrafish that ultimately impacted central nervous system health.^6^ Beyond these physiological effects, smaller micro- and nanoplastics can transport pollutants at higher abundance per mass than larger particles due to their increased surface area to volume ratio;^7,8^ the transport effects may be exacerbated by the fact that these particles are small enough to cross biological barriers. Due to the ecological and human health implications related to the size of micro- and nanoplastic exposure, it is critical to characterize the size fractions present in natural systems in order to predict their potential for harm.

Many freshwater and marine studies report or predict that there will be an increase in the number of particles as particle size decreases and that these smaller particles likely originate from degradation of a larger-sized plastic.^9−11^ In natural sample studies, size distributions have been observed either through collection of particles on sieve/filters with varying mesh sizes and/or with visual microscopy imaging that allows for direct measurement of particle dimensions or FTIR microscopy where pixel size is used to estimate particle size.^12,13^ However, quantification of the smaller size fractions has been difficult with current enumeration approaches.^14−16^ For example, a round robin study with known plastic samples showed a 32 ± 16% recovery for particles in the 3–20 μm range.^17^

Because of the challenges in isolating and analyzing small micro- and nanoplastics in natural samples, efforts to model the distribution of plastics within natural systems have been applied to predict the potential abundance of small plastic particles. This model typically applies a power law relationship to data in measurable size ranges^7,18^ and is sometimes extrapolated to ranges more difficult to measure in all samples.^19−21^ The power law predicts an increasing abundance of smaller particles, with an exponent of ∼3 often occurring in natural particle studies, indicating that larger particles fragment into smaller particles in three dimensions. Deviations from the power law are interpreted as resulting from additional inputs of plastic particles, *in situ* removal processes, or changes in the fragmentation processes.^18,22^ A shift in the power law exponent away from 3 is interpreted as a change in the dimensionality of fragmentation.^18,22^ In practice, the exponent is often calculated as the negative slope from a plot of the log of the size fractions (as the *x*-values) to the log percent abundance or normalized abundance within the size fraction (as the *y*-values).

While the initial intention of the power law was to reveal possible sources and sinks within measured size fractions,^18^ researchers have begun to apply the model to predict abundances of unmeasured size fractions. Such predictions have been used to rescale field and toxicology studies to the same microplastics size ranges for risk assessment and inter-ecosystem comparisons.^19,21,23^ Kooi and Koelmans applied the power law model of analysis to 19 studies that varied in sampling locations and determined an average exponent value of 1.6^11^ while a larger study that included marine and freshwater surface waters and sediments found that the exponent value for these combined samples was 2.68.^7^ The variation in the exponential values in these different studies as a function of the number of samples included suggests that the assumptions made during fitting data may influence the model and, thus, our ability to predict abundances of smaller particles. Further concerns about the power law model are raised when considering sampling near urbanized cities where the power law does not describe the plastic size distribution as larger-sized plastics are in greater abundance compared to smaller particles.^24^

The work presented here investigates size distributions and polymer compositions of plastic particles in western Lake Superior and the adjacent Duluth-Superior Harbor, which contains the estuary of the St Louis River and is an urbanized setting relative to most of the Lake Superior coastline. This study provides a variety of novel angles including sample sites that are limnologically connected and size fractionation in freshwater, a generally understudied venue. We experimentally quantify size fractions smaller than have been previously investigated in the Laurentian Great Lakes.^25−29^ This is the first study to apply concurrently measured size, morphology, color, and polymer data on microplastic particles from 50 to 300 μm in size along a river to Great Lakes transect. It also investigates the size distribution at each sampling site relative to the power law relationship and the plastic polymer composition, thus revealing important connections between site-specific plastic distributions and anthropogenic, geographic, and limnological influences.

## Materials and Methods

### Sampling Sites

Samples were collected from the Duluth-Superior Harbor on September 21, 2021, via the *R/V Kingfisher* and far-western Lake Superior on August 3–4, 2021, using the *R/V Blue Heron* (Supporting Information Figure S1). Weather on September 21, 2021, in the harbor was partly cloudy to sunny with generally calm conditions. The Duluth International Airport weather station reported 0.55 in. of rain in the 24 h preceding sampling. Weather on August 3–4, 2021, was generally calm and partly cloudy. The Duluth International Airport weather station reported moderate winds and no rain on these sampling days or the 24 h period before the cruise.^30^ Harbor stations (A, B, C, and K) are expected to be heavily influenced by the Duluth-Superior metropolitan area (population ∼113,000) as well as input from the St. Louis and Nemadji Rivers.^31^ Lake sites (4, 2, 7, and 12, in order of sampling) were chosen for their variability in possible pollution sources and their limnological characteristics. Station 4 is an open-water station off the Duluth entry to the Harbor.^28^ Stations 7 and 12 are nearshore locations expected to be affected by river runoff and erosion from clay cliffs along Lake Superior’s southern shoreline. Station 12 is closest to the Superior, Wisconsin, entrance to the harbor, while station 7 is near Herbster, Wisconsin. Station 2 is the deepest station with a water column depth of 257 m.

### Sampling Strategies—Cascade Filter Tower and McLane Pump

This study employed two different sampling methods: surface water pumping coupled with cascade tower filtration, targeting 1 m water depth, and *in situ* pumping using a Large Volume Water Transport System (WTS-LV, McLane Research Laboratories, Inc.), targeting 2 m water depth. Total microplastic counts (summation of the ∼50–100 μm, ∼100–300 μm, and 300 μm–5 mm fractions) from samples collected at the same stations by the two pumps did not show significant differences via a two-tailed, pairwise *t* test (SI, Figure S2). To further supporting our choice of pumping at a 1 to 2 m depth, a study of surface and subsurface water concentrations in Lake Michigan and some of its estuarine waters using net tows showed that water column concentrations are overestimated by using surface net tows.^32^ Previous work in Lake Superior shows that microplastic particles collected by Manta net do not have consistently low densities and that pump samples at 2 m depth actually had higher microplastic concentrations than seen in Manta net samples from the same Lake Superior location.^27,28^ We suspect that there is mixing of the topmost layer of the water column with the air–water interface in Lake Superior and other large lake systems, which explains these trends.^27,28^ Additional descriptions of sampling choices are in the SI.

The cascade tower consisted of three metal mesh sieves (300, 106, and 45 μm) and a 5 μm nylon mesh held in place by metal collars. Water from 1 m below the surface was filtered through the sieves using a diaphragm pump (Ingersoll Rand Aro double diaphragm pump, model PDO2P-APSPTA) and polyethylene tubing to draw water up to the deck of the boat and through the sieves at a flow rate of ∼4 L/min. The volume of the filtrate was measured using a stainless-steel bucket with a bottom spigot and a flow totalizer (Carlon, Model: 750JLP RS) with 100 to 500 L of water being filtered onto the mesh sieves over 1 to 2 h per station. 10 to 20 L of the water that had gone through the metal sieve stack was collected onto the nylon 5 μm screen, as this screen was prone to clogging or failure at higher volumes (see Minor et al., for further details on collecting the 5 to 45 μm size fraction).^33^ After the water was sieved, each metal sieve was rinsed/backflushed into a clean mason jar (previously combusted at 450 °C) with Milli-Q Ultrapure water and forceps were used to remove all visible particles from the sieve into the jar. Between sampling sites, the sieves were further backflushed with Milli-Q water to clean them and covered in aluminum foil to prevent contamination. A new nylon mesh was used at each sampling location.

McLane pumping was performed at the Lake Superior stations as previously described in Fox et al.^27^ Stations 2, 4, and 7 were sampled at a 2 m depth. Prior to sampling, the McLane pump was backflushed with 5 L of Milli-Q water to clean the pump and each pump was outfitted with 300, 100, and 50 μm nylon filters (previously rinsed by sonication with Milli-Q water, with this rinsing step performed a total of three times). The target sample volume was set to 500 L at a flow rate of 4 L/min. The pump was then deployed at the appropriate depth using the research vessel’s A-frame. After sample collection, the pumps were returned to the surface and the filters were removed using forceps and transferred to clean, prelabeled glass mason jars with ∼100 mL of Milli-Q water added to prevent drying.

### Sample Processing and Analysis

Samples were processed as previously described.^27,28^ Briefly, samples were dried in an oven at less than 90 °C and then oxidized using Fenton’s reagent. A saturated sodium chloride solution (∼5 M, ∼1.2 g/mL) was then used to remove the higher-density mainly mineral material (such as sand and clay) that survived the oxidation process. The choice of saturated NaCl solution is a trade off between the loss of more dense plastics such as PVC^28^ and the retention of fine inorganic particles such as clays that complicate FTIR microscopy analyses of natural-water samples.^34^ The supernatant from density fractionation, and the plastic particles within it, was then filtered onto aluminum oxide Anodisc filters (Cytiva Whatman Anodisc filter membranes, 0.2 μm pore size) for the 106 and 45 μm size fractions, and onto mixed cellulose ester filters (Millipore, 0.45 um gridded MCE) for the 300 μm size fraction, and nylon 0.45 μm Millipore HNWP membrane filters for the 5 μm size fraction. The 106 and 45 μm samples were scanned via a Nicolet Continuum Infrared Microscope, while the 300 μm size fraction was analyzed via visual microscopy and melt testing, with isolatable particles further characterized by ATR-FTIR. The 5 μm size fraction was rinsed and resuspended off the filter with approximately 5 to 7 mL of Milli-Q water and sent for staining and flow cytometry analysis at the Bigelow Laboratory for Ocean Sciences.^24^ See the SI for further details on analyses.

### QA/QC—Blanks and Controls

During cruises, all scientific personnel wore cotton or wool to minimize contamination from synthetic textiles; however, life preservers and other work vests necessary on deck were present and contained synthetics. Lab coats and cotton clothing were worn during the sample processing and analysis. All glassware used was combusted at 450 °C for 8 h prior to use and covered in aluminum foil as much as possible during sample processing to limit possible contamination.

Two types of method blanks were collected, as two different pumps were used in this study.

The cascade pump blank consisted of <0.8 μm filtered deionized water, which was placed in an HDPE barrel and then run through the diaphragm pump, sample tubing (also PE), and the metal sieve stack. Prior to the collection of this blank, the tubing was rinsed with 10 L of Milli-Q water and the sieves were cleaned with hot soapy water, scrubbed with a natural sponge, and rinsed with Milli-Q water. The cascade pump blank consisted of 179 L of the <0.8 μm water pumped through the apparatus at an average flow rate of 6.6 L/min. The particles collected on the sieves were immediately filtered onto Anodiscs and thus represent a field sampling blank only.

As the McLane pump is an *in situ* submersible pump that vacuum-filters the samples, it presents challenges in obtaining a deionized water-based blank. Instead, a 5 μm mesh filter was placed in the uppermost filtration slot, which allowed Lake Superior water of less than 5 μm to pass through when it was submerged in the lake. The “microplastic free” water then passed through 100 and 50 μm mesh filters, and any >5 μm particles collected in the lower two filter slots were considered to be contamination from the pump and/or subsequent sample processing. Two McLane pump and processing blanks (Mc-A and Mc-B) were performed at a 2 m depth at different locations in open-water Lake Superior. For the first blank (Mc-A), the sampler was submerged at 2 m and allowed to pump 172 L of water. For the second blank, Mc-B, the pump was again submersed at a 2 m depth and 86 L of water was filtered. Both blanks had a flow rate of 4 L/min. The McLane blanks underwent all sample processing steps (resuspension, oxidation, density extraction) and are thus full field sampling plus processing method blanks.

The cascade pump blank was analyzed by the Nicolet Continuum Infrared Microscope (μFTIR) using the same analytical parameters as the samples but scanning 10% of the filter. The McLane pump collected field plus method blanks were analyzed by μFTIR on a Bruker Lumos II instrument with particle identification using the software package Purency. This combination of instrumentation and analysis provides microplastic size, as well as polymer information. Based upon the relatively low contribution of particles per liter and the mismatch of polymer, color, and morphology characteristics between the particles in the blanks and samples, no blank corrections to the field data were performed. More detailed descriptions of the blanks, including the results, are provided in the SI.

Positive controls were used to evaluate recoveries in the sampling, sample processing, and analyses. Recovery from the cascade sieves was tested using visual microscopy and a standard consisting of PE spheres (600–710 μm, Cospheric, Product ID: CPB-0.96), PVC fragments (250 μm, bought from Goodfellow, manufactured by Ineos, product code CV316010), and PMMA spheres (85 μm, Goodfellow, 729-305-51), all used as obtained from the supplier and added to Milli-Q water. The Thermo μFTIR positive control standard was directly filtered onto an Anodisc with 10% of the filter scanned and counted. This test was performed in triplicate (i.e., three separate filters were prepared). Recovery was tested of PA 55 μm powder (Goodfellow, AM306055/1), PMMA 85 μm spheres (Goodfellow, 729-305-51), and MDPE (250 to 350 μm, Goodfellow, EV306010). Additionally, positive control was performed propagating across all the steps (filtering, oxidation, density extraction, and μFTIR). All recovery results with discussion^35−40^ are reported in the SI.

### Data Analysis and Power Law Modeling

Size distribution data was modeled using a power law (eq 1) reconfigured to eq 2;^11^*x* is the “bin” size in microns, and *y* is the normalized abundance (e.g., percentage). To model the size distribution data simply and for the easiest comparison with literature data, we applied the power law to the longest dimension of each particle based upon μFTIR pixel size or to the smallest particle size expected to be retained on the sieves (for the 5–50 and >300 μm size range). The particles are thus generally sized in 50 μm increments (e.g., 50–99 μm is listed as the 50 μm bin); the smallest size class (5 μm bin) reflects 5–50 μm, and the largest size class (300 μm bin) represents 300 μm to 5 mm.
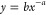
1
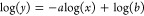
2

The log-transformed data underwent linear regression analysis, where the slope (*a*) can be related to slopes from microplastic size-spectrum analyses in other aquatic systems; the linear regressions were performed in Excel. Principal component analysis (PCA) was applied to the data to determine relationships between polymer type, metal sieve size, and location. Polymer composition counts were normalized to total particle count for each size class at each station. PCAs were concurrently calculated with their corresponding ordination analyses, indicating which polymer is influencing variation. PCA and ordination were completed using the FactomineR Package in the R statistical computing program.^41^ All other figures created using ggplot2 package.

## Results and Discussion

### Microplastic Counts and Morphology/Color Analysis

**Figure 1:**
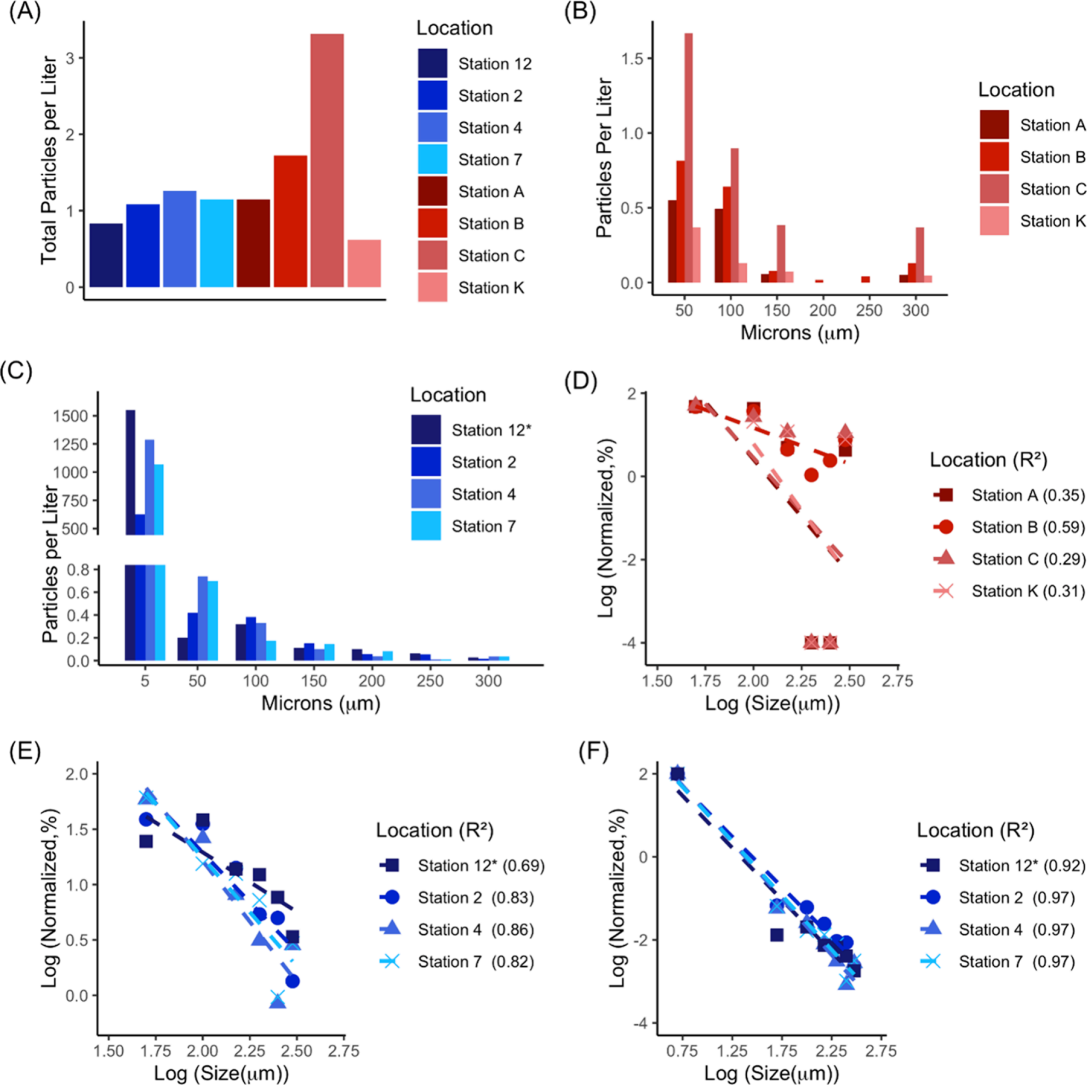
(A) Microplastics in surface water (1 to 2 m depth) summed across the sampled size ranges (thus 50 μm to 5 mm) per station location. (B, C) Size fraction distribution at the harbor and lake sampling locations. Note that for the *x*-axis, “50”, refers to the 50–99 μm size fraction; “100” to the 100 to 149 μm size fraction, etc. (D, E) Size distribution via power law of harbor and lake surface water at each station. When no plastics were found in a sample size range, the *y*-value is given as −4 (i.e., 10^–4^ counts). (F) The power law applied to lake surface sample data and extended to the 5 μm size fraction using flow cytometry data from Minor et al.^33^ Lake stations in A, C, and E, and F consist of averaged values of cascade and McLane pump surface water numbers (see Tables S1 and S2). * indicates the lake station for which there are numbers from cascade sampling only.

Microplastic particle counts per liter of water (MP/L) from 1 m depth (Figure 1A and SI Table S1) ranged from 3.32 MP/L at station C, a harbor site, to 0.62 MP/L at station K, also a harbor site. Sites A and B, the remaining harbor stations, had values of 1.15 and 1.72 MP/L, respectively. These river-harbor values are lower than seen in the most urbanized samples from a Dutch river study of particles >20 μm in size (8.4 to 11.5 MP/L) but within the range seen in these rivers (0.16 to 11.5 MP/L) in a study that used similar sampling and analysis techniques but a different oxidation and density separation protocol.^13^ The values are considerably lower than a Wisconsin, USA, river study targeting the >10 μm range and using Nile Red staining, epifluorescence microscopy, and melt testing for microplastic identification, which found average particle concentrations in the 50 μm to 5 mm range for upstream, urban, and downstream locations to be ∼150, ∼490, and ∼250 MP/L, respectively.^42^ In the lake, values at 1 m depth ranged from 0.83 at station 12 to 1.43 at station 2 (Table S1), within the range seen previously for the same size ranges in western Lake Superior (0.25 to 1.9 MP/L).^27^ That harbor stations C and then B have the highest concentrations of plastic particles may be explained by the proximity to urbanized areas (including a wastewater treatment plant and harbor facilities) as seen in other studies.^13,43,44^ However, it is also important to note that station C sample analysis varied from that of the other samples. Harbor samples had high clay contents and thus were split into aliquots and filtered onto separate Anodiscs. For the station C 106–300 μm size fraction, one Anodisc was unable to be scanned with μFTIR due to the thickness of the sample and the amount of clay deposited. Therefore, the value reported for the station C 106–300 μm sample was obtained by doubling the counts obtained from the other Anodisc onto which half the sample volume had been filtered (see the SI for more details).

Morphology and color were compared across harbor and lake samples taken from 1 m depth. In the harbor, the majority microplastic morphology was fragments (55% of identified microplastics) followed by fibers (29% of identified microplastics). Fibers were especially dominant in the >300 μm size range, where they were 87% of the identified microplastics (SI Figure S3). This dominance of fibers in >300 μm microplastics has also been seen in subsurface water samples in river to nearshore lake transects in Lake Michigan and in all water samples from the Milwaukee Outer Harbor and Lake Michigan sites in that same study.^32^ Microplastic particles in our harbor samples were predominantly translucent or white (66%) but also exhibited a wider range of colors than particles in the lake (SI Figure S3). The percentage of particles found to be translucent or white was very similar to that found for >300 μm microplastic particles in in surface waters from smaller Minnesota lakes (65%)^45^ and for >125 μm anthropogenic particles in nearshore Lake Ontario (∼50%).^44^ In the lake, the predominant morphology was again fragments (54% of the microplastic particles) but fibers were less abundant (7% of the microplastic particles), perhaps because larger particles themselves were also less abundant. Many more lake than harbor particles were of unknown morphology, which were designated as unknown because of the challenges in seeing the particles within the clay matrix that remained after density separation and in part because of the size of these particles. Previous studies of larger (>500 μm) microplastic particles in Lake Superior collected by Manta net found fibers as the predominant morphology (67%) with fragments present at 23%.^26^ Microplastic particles in the lake, like those in the harbor, were predominantly translucent or white (72%, SI Figure S3). The higher proportion of white or translucent particles in the lake relative to the harbor follows similar trends to those seen in studies of micro and macroplastic particles (0.2 mm to 15 cm) in marine surface waters, where larger proportions of white particles were seen in both smaller particles (<5 mm) and offshore particles.^46^ The authors of this marine study attribute these trends to extensive weathering of the smaller and more offshore particles.

Our harbor sites are impacted by runoff from the St. Louis River, input from a water treatment plant, and port activity that could contribute to microplastic counts. Yet, the microplastic concentrations (median_harbor_ = 1.44 MP/L; median_lake_ = 1.20 MP/L), colors, and morphologies found in the lake and harbor are fairly similar. These similarities could indicate that there is extensive homogenization of the lake and harbor water due to seiche activities in this freshwater estuary system. However, the difference in size distributions and polymer compositions between the harbor and lake samples is a counter-argument to hypothesized homogenization. Our data instead suggests that total microplastic particle count, morphology, and color may not be the best indicators of microplastic sources and interactions in the aquatic environment.

### Size Fraction Analysis and Power Law Fitting

While the overall counts, colors, and morphology are similar, differences between the harbor versus lake samples appear when we compare the size distribution of the plastic particles (Figure 1B,C; SI Tables S1 and S2) and attempt to fit these to a power law distribution (Figure 1D,E and SI Figure S4). Most notably, the harbor stations differ from the lake samples in having a larger portion of microplastics in the >300 μm size fraction and few particles in the 200 and 250 μm size bin. The harbor samples, thus, are not well described by the power law (with *R*^2^ values ranging from 0.29 to 0.59). If the power law is really applicable to microplastic degradation in aquatic systems, the size distribution at the harbor stations indicates either a recent source of larger plastic inputs or a removal mechanism for the intermediate-sized microplastics.^18^ We, therefore, hypothesize that proximity to sources (e.g., river inputs, major port facilities, and population centers) results in the particles having experienced a shorter residence time in water and thus less of an opportunity to break down into smaller particles. For the four lake samples, the power law seems to describe the size distribution reasonably well (with *R*^2^ values ranging from 0.69 to 0.86, and α (see eq 1) ranging from 1.1 to 2.2). Although all lake sites show some concavity in size distribution, in distinct contrast to the somewhat convex shape of the harbor distributions. The concave distributions indicate potential inputs in the intermediate size ranges or potential preferential removal of the largest and smallest size ranges, perhaps through fragmentation and hydrodynamic transport.^47^ Note also that zooplankton grazing at open-water Lake Superior sites might also be responsible for particle decreases in the 50–100 μm size range as the lake’s algal biomass is overwhelmingly in particles less than 80 μm.^28^ However, it is also worth noting that our analysis of control samples indicates that particles ∼55 μm in size are likely undercounted (see the SI for further details).

We also quantified 5 to 45 μm size fraction samples collected from the cascade tower filtrate and analyzed them using flow cytometry.^33^ When adding the smallest size fraction to the lake samples, the power law appears more applicable (with *R*^2^ from 0.92 to 0.97, Figure 1F and SI Figure S5) and the exponent also shifts dramatically, with α ranging from 2.4 to 2.7. This suggests that the power law is sensitive to the size ranges used to predict trends and indicates that predicting smaller size ranges from larger ones^19,20^ may not work in many cases. In using the power law from Figure 1E and extrapolating to total particles from 5 to 5000 μm (using the previously reported correction factor equation^7^), the power law predicts particle concentrations from 1.3 to 12 particles per liter, while our flow cytometry counts in the 5 to 45 μm range alone averaged 1100 particles per liter.^33^ An estimated threshold for risk assessment based upon a 5% species affected criterion and food dilution as a mechanism has been estimated as 11 to 521 particles (in the size range 1–5000 μm) per L^20^ or averaging 547 particles per L.^48^ Yet, the difference seen in predicted (1.3 to 12 particles per liter) vs measured (600 to 1600 particles per liter) particle numbers in our 5 to 5000 μm samples indicates that using the power law to predict microplastic counts is problematic because it is highly sensitive to the range of sizes measured in the sampling and leads to significant underprediction of the concentration of smaller plastics.

### Polymer Composition

**Figure 2:**
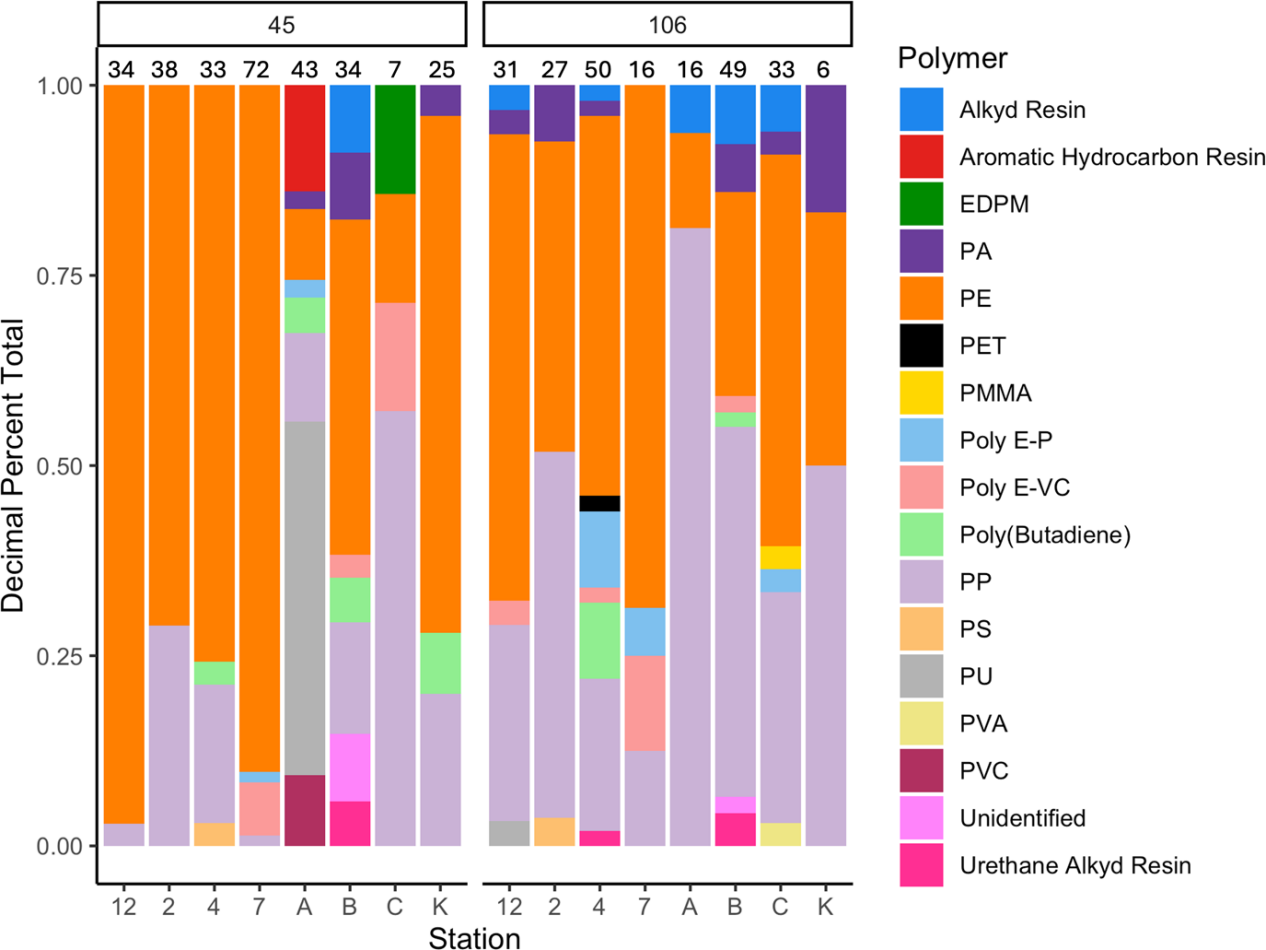
Compositions obtained from μFTIR analysis grouped by metal sieve size in μm used for sample collection from a cascade filtering apparatus (1 m depth water samples). In each size fraction, lake samples are the numbered stations, while letters indicate the harbor stations. Numbers above bars are the number of particles that were counted and identified in each size/station sample. Abbreviations: EDPM, ethylene propylene diene monomer; PA, polyamide; PE, polyethylene; PET, polyethylene terephthalate; PMMA, poly(methyl methacrylate); poly E-P, poly(ethylene propylene); poly E-VC, poly(ethylene-vinyl chloride); PP, polyprolylene; PS, polystyrene; PU, polyurethane; PVA, poly(vinyl alcohol); PVC, polyvinyl chloride.

Like the size distributions discussed above, the polymer composition of the samples showed interesting trends with the sample location (Figure 2). The harbor samples as a group have a wider variety of polymer types than the lake stations do, perhaps because of the large number of potential plastic sources to the urbanized harbor. The polyolefins that are most widely used (i.e., polyethylene and polypropylene) make up a large portion of the particles collected in both harbor and lake samples. However, polyethylene (PE) and polypropylene (PP) particles make a larger proportion of the particles in the lake, perhaps because these polymers are less photodegradable than other microplastics^49−51^ in the clearer lake waters, which are also more remote from urban plastic sources. The lake distributions could also reflect the lower mechanical stability of PP and PE, which would thus make such particles more likely to fragment,^52,53^ and thus yield higher particle numbers in the longer residence time waters of the open lake. However, station 4, a lake station, shows the greatest variability in polymer types, perhaps because of its proximity to Duluth and its limnological connections to the harbor.^54,55^ Station A, the most upstream of our samples, shows the highest polymer variation within the 45 μm fraction and contains very little PE and PP within that size fraction, although the 106 μm size fraction contains mainly PP.

When examining the polymer variability based on size fraction, the smaller size fraction (45 to 106 μm) in lake samples has fewer polymer types (Figure 2). Across all samples, with the exception of site A, PE and PP comprise the largest portion of the smaller particles. The prevalence of PE and PP in the smaller particles, and especially in the lake samples, is, as mentioned above when comparing lake vs harbor samples, most likely due to the low mechanical strength of both polymers, such as tensile strength (4.1–15 MPa (for low-density PE—LDPE) and 29–35 MPa (for PP)). PE and PP are thus more likely to break down to smaller sizes at a faster rate in comparison to higher tensile strength polymers such as polyamide (PA), poly(methyl methacrylate) (PMMA), and polyvinyl chloride (PVC) (82.7–90.3, 48–76, and 56.5 MPa, respectively).^53^

**Figure 3:**
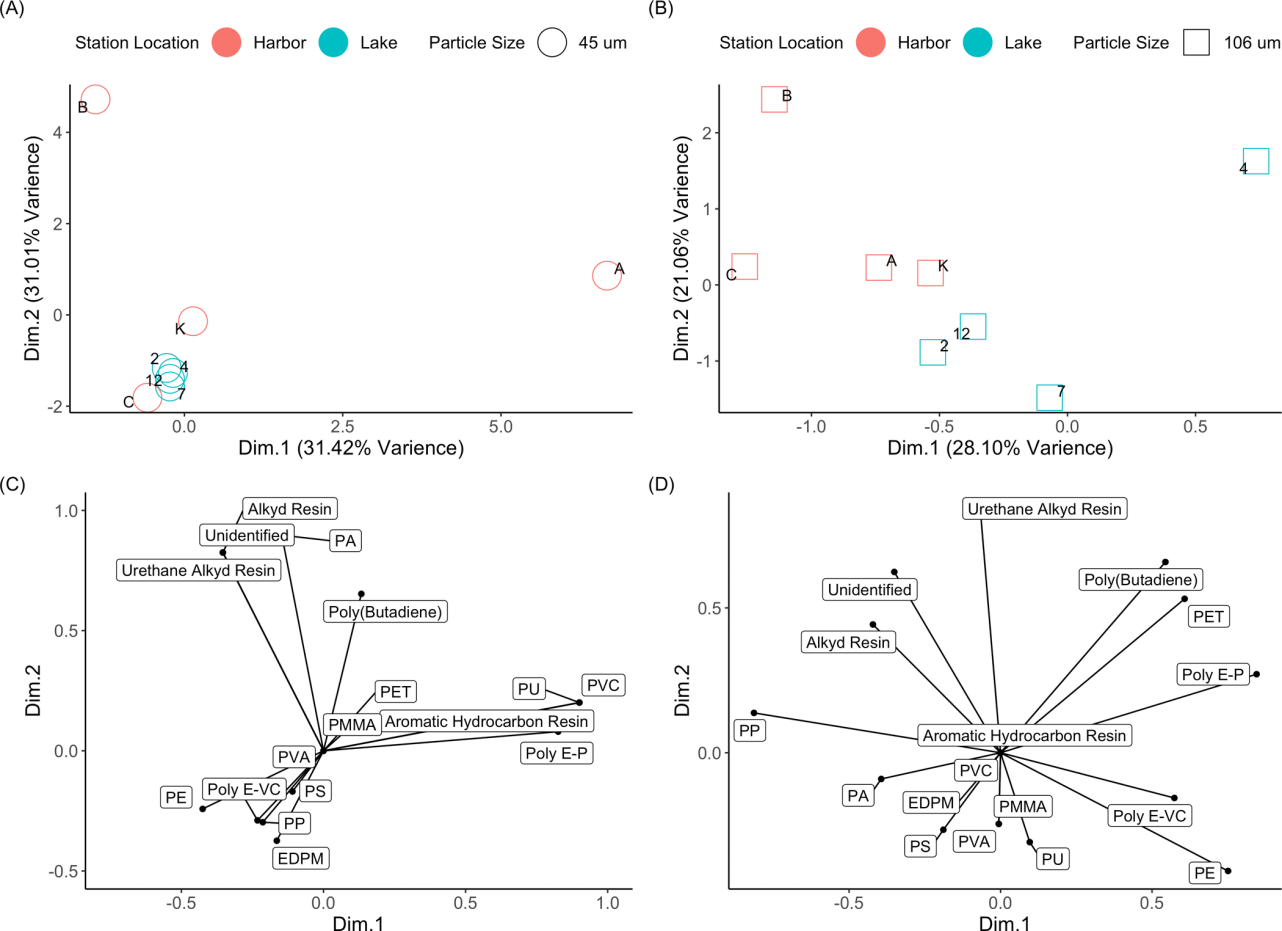
Principal component analysis (PCA) (A, B) with corresponding ordination plots (C, D) of each metal sieve fraction (cascade sampling) with respect to polymer composition abundance. Plots on the left side (A, C) represent the 45 to 106 μm fraction, while the right sides B and D represent the 106 to 300 μm fraction. Dim 1 and Dim 2 are principal components 1 and 2.

PCA of the polymer composition of the size fractionated samples confirmed that polymer distributions are related to the sampling location (Figure 3). In the 45–106 μm fraction (Figure 3A), all four lake stations plot in a small portion of the overall principal component 1 vs principal component 2 space, while the harbor stations appear much more diverse in composition. As seen in ordination (Figure 3C), the assortment of different polymers separates the harbor stations among themselves, while the lake samples are defined by a large proportion of PP and PE. In particular, harbor stations A, B, and C define the majority of the overall variation of all the stations within the 45–106 μm fraction. This pattern of clustering within the lake and harbor samples is not displayed in the 106–300 μm fraction, which instead shows the lake samples separated from the harbor samples along principal component 1 (28% of the total variance, Figure 3B). The ordination (Figure 3D) shows that the variance along PC1 is characterized by polyethylene terephthalate (PET), polybutadiene, poly(ethylene:propylene) (polyEP), and PE along the positive axis and PP, PA, and polystyrene (PS) along the negative axis. Samples are separated along principal component 2 (21% of the variance) by varying proportions of many polymers on the negative axis, and alkyd resins, PET, and polybutadiene on the positive axis. Alkyd resins, which appear to characterize station B in both size fractions, are commonly used in surface coatings, such as paints used on buildings, roads, and ships.^56^

### Summary and Implications

To summarize our results from western Lake Superior and the Duluth-Superior harbor, canonical approaches to characterizing microplastic distributions via total plastic counts, morphology, and color may not capture information that allows elucidation of microplastic sources and environmental fate. Specifically, overall microplastic (50 μm to 5 mm) counts ranged from 0.62 to 3.32 MP/L and did not show major differences between the harbor vs lake sites, although the two sites with highest concentrations were in the highly urbanized portion of the harbor. Morphology and color distributions were also similar for the lake and harbor samples, although harbor particles included a wider range of colors. Rather, distinct differences in the size fraction and polymer composition revealed important site-specific trends. That is, particle size distributions, in contrast to total counts, appeared quite different between the harbor and the lake. The harbor stations deviated from a power law distribution with larger proportions of the total plastic counts in the >300 μm size range and few particles in the 200 and 250 μm size ranges, perhaps due to proximity to microplastic sources and lower water residence times for the harbor vs the lake. The lake samples from 50 μm to 5 mm indicated deviation from the power law due to greater than expected proportions in the intermediate (100 to 200 μm) size range. The polymer composition also varies with particle size and sampling location, with the lake sites being characterized by PE and PP in the 50–100 μm size range, while the harbor sites were much more diverse in polymer composition. This may be due to the relatively low mechanical strength of PE and PP, which would allow them to be fragmented into smaller particles more easily and perhaps be more prevalent in the more reworked particles of the open lake. For the 100–300 μm size range, the lake sites were distinguished from each other based on PP vs PE, poly(butadiene), PET, and polyEP while the harbor samples were distinguished from each other based on PP and PA vs alkyd resins.

This work also demonstrated that the power law size distribution and deviations from it appear to provide key information about the relative importance of recent sources to a plastic particle pool and on potential removal mechanisms in aquatic systems. The complexity of polymer composition, likewise, appears to be a function of proximity to sources, with more reworked open lake samples showing simpler polymer distributions dominated by PP and PE. Addition of the 5 to 45 μm size class to the power law distribution of lake samples dramatically improved the fit and significantly increased the power law exponent at each station. The α changed from 1.1–2.2 to 2.4–2.7 when the 5 to 45 μm size range was included. It is important to point out that such shifts in α challenge assumptions that the use of the power law can be used to rescale microplastic abundance data collected at one size range to another or to extrapolate from the easier-to-measure large particles to abundances at smaller size ranges such as nanoplastics. Our work shows that the model is susceptible to error depending on the size binning chosen for the model fits. Additionally, it fails to predict the appropriate microplastic abundance of small-sized particles in Lake Superior. This highlights the need for a detailed size characterization across representative aquatic systems. Based upon our data, the power law does not appear to be an appropriate model for risk assessment applications.

## Supporting Information Available

Sampling site map and coordinates, additional methods for identification and characterization of microplastics with FTIR, and statistical validation of analysis choices (PDF)
