# Supplementary material for: Size Distributions of Microplastics in the St Louis Estuary and Western Lake Superior
Source: Environ Sci Technol. 2024 May 2;58(19):8480–9. doi: 10.1021/acs.est.3c10776 (PMC11097629; doi:10.1021/acs.est.3c10776)
Supplement: Supplementary file 1 — es3c10776_si_001.pdf [file es3c10776_si_001.pdf]

Supporting Information

Size Distributions of Microplastics in the Saint-Louis Estuary and Western Lake Superior

Ariyah Thomas[a1], Joseph Marchand[a1], Guenter D. Schwoerer[1], Elizabeth C. Minor[1,2]*, Melissa A. Maurer-Jones[1]*

[1]Department of Chemistry and Biochemistry, University of Minnesota, 1038 University Dr., Duluth, Minnesota 55812, United States

[2] Large Lakes Observatory and Department of Chemistry and Biochemistry, University of Minnesota Duluth, 2205 East Fifth St. Duluth, Minnesota 55812, United States

* corresponding authors (eminor@d.umn.edu and maujones@d.umn.edu)

[a] co-first authors

Summary of SI Content

Pages: 13

- Includes additional figures, tables, method details, and results

Figures: 5

- Fig. S1. Sampling sites
- Fig. S2. Comparison of pump sampling strategies
- Fig. S3. Morphology and color of plastics results
- Fig. S4 and S5. Individual power law plots

Tables: 2

- Microplastic abundances for cascade filtration (Table S1) and McLane pumps (Table S2)

Sample Site Information:

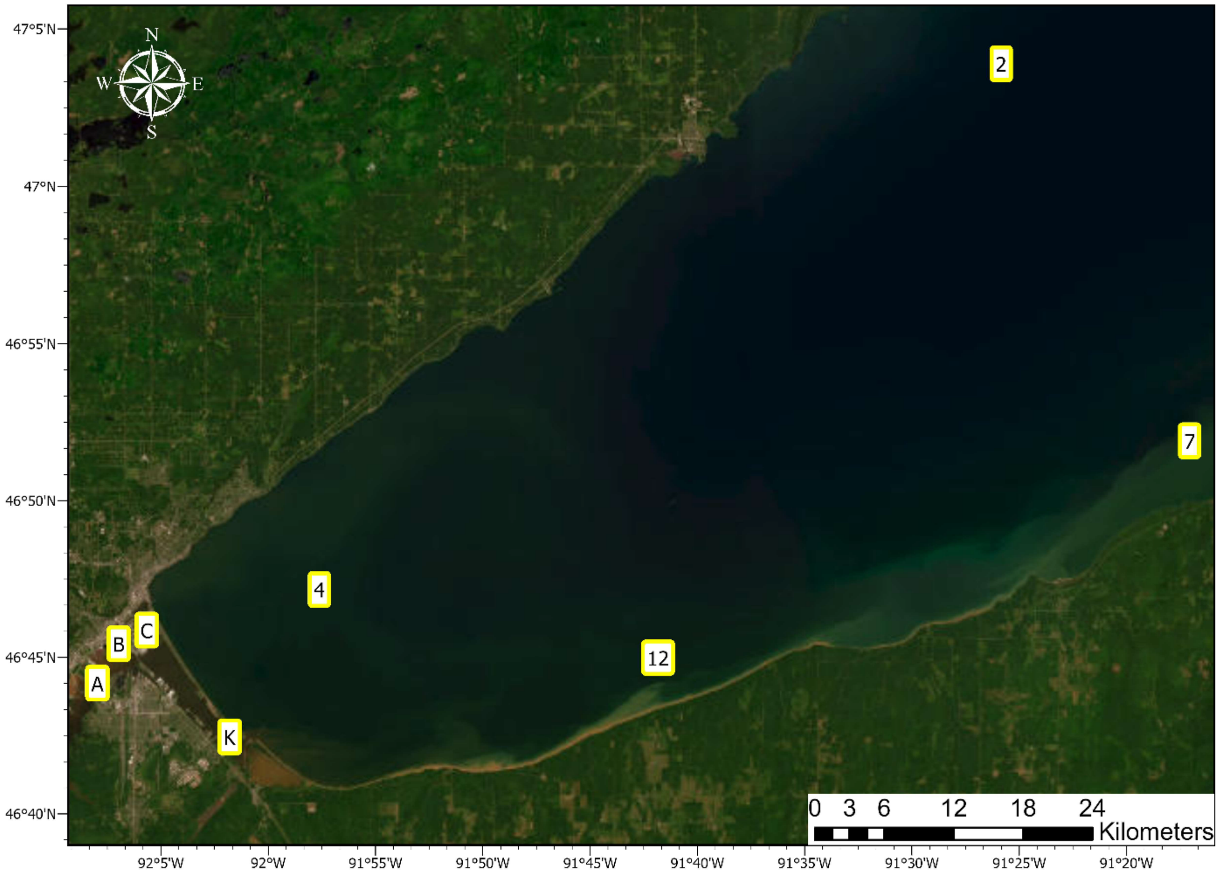

Figure S1. Map of the 4 harbor stations (designated with letters) and 4 lake stations (designated with numbers). Station A (Inner estuary): 46°44.15'N, 92°07.96'W, Station B: 46°45.43'N, 92°06.97'W, Station C (Dock, Harbor): 46°45.86'N, 92°05.68'W, Station K (Nemadji River): 46°42.51'N, 92°01.77'W. B), Station 2: 47°03.9'N, 91°25.9'W, Station 4: 46°47.3'N, 91°57.8'W, Station 7: 46°52'N, 91°17'W, and Station 12: 46°45'N, 91°42'W. Note: In-harbor sites were also studied in Hendrickson et al, 2018[1] and used the same station labels. The lake sites were studied in Minor et al 2019[2] and have the same station names. Sources: ESRI, DigitalGlobe, GeoEye, I-cubed, USDS FSA, USGS, AEX, Getmapping, Aerogrid, IGN, IGP, swisstopo, and the GIS User Community.

Comparison of Counts from McLane and Cascade Sampling Methods

This study used two different sampling methods, cascade tower filtration at 1 m water depth and McLane *in situ* pumping at 2 m water depth. The cascade tower is more amenable to small boat sampling, as was used in the harbor. The McLane pump can collect particles while submerged in the water down to depths of hundreds of meters. In addition to allowing the possibility of deepwater sampling, McLane pumps also minimize sample contact with the air during filtering and are therefore less likely to show ambient contamination from dust, personal protective gear and other materials present on a ship deck. Both approaches minimize on-deck handling of samples relative to Manta net towing and subsequent rinsing of the sample into the cod end. Previous water sampling in Lake Superior shows 2 to 3 orders of magnitude higher particle concentrations obtained via McLane pump at 2 m depth (for >300 µm particles) than obtained

via Manta net (>333 µm) at the same site and roughly the same time.[3] Comparison of net deployments at different depths in Lake Michigan do not find significant differences in particle concentration at the air-water interface vs at depth in the water column.[4] Total microplastic counts from samples collected at the same stations by the two pumps did not show significant differences as determined by a two-tailed pairwise t-test ($p=0.799$). Size distributions do not show trends as a function of pump choice (SI Fig. S2)

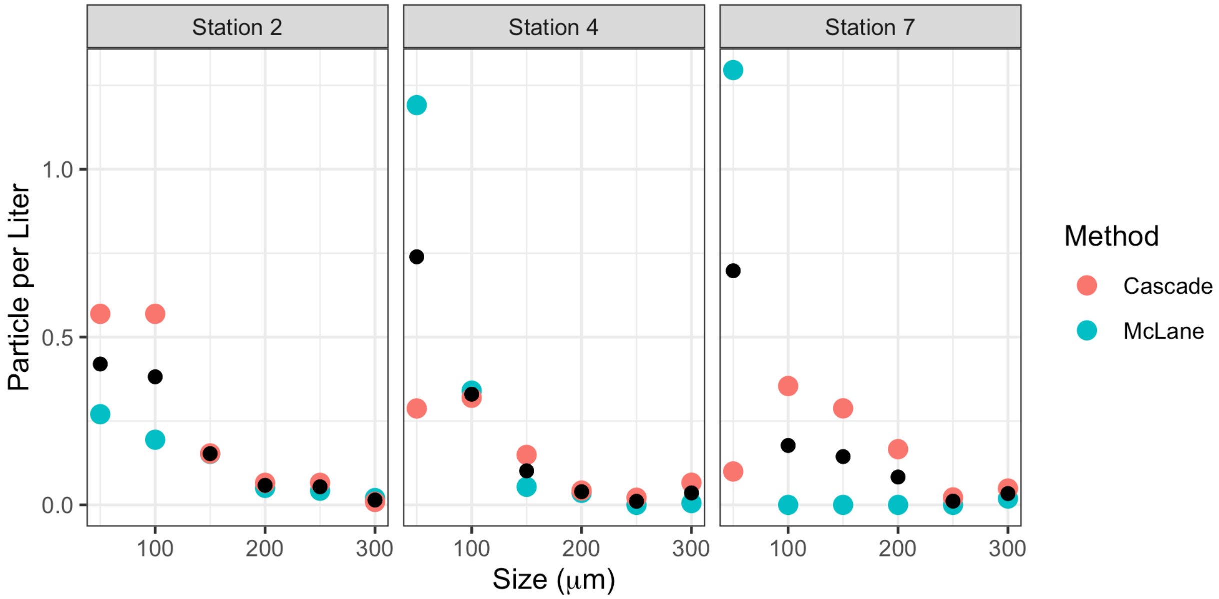

Figure S2. Cascade (Red) and McLane (Blue) samples were taken approximately concurrently at each sample location. Particles per liter are plotted per 50 µm size bin, per lake station, excluding station 12 where no McLane data is available. Black dots represent the average value between the sampling techniques used in the manuscript for size spectrum analyses. No consistent bias observed with one sampling method versus the other so the average values were reported.

Visual Microscopy and ATR-FTIR

All samples >300 µm were inspected with visual microscopy (10 to 40x) using a 3.5X-90X LED trinocular zoom stereo microscope with a 10-megapixel digital camera (AmScope, Irvine, CA). The samples were filtered onto MCE 45 µm gridded filters that were kept in clean (previously combusted) glass Petri dishes at all times though the covers were removed during analysis. Each 3.1 x 3.1 mm square of the gridded filter was inspected for potential plastic particles. The suspected plastic particles were photographed, and pressure tested using forceps, and if the particle did not crumble, then a melt test was performed by touching the particle with a hot needle (as in Hendrickson et al., 2018)[1]. The particle was identified as plastic if the particle was melted by the hot needle. Microplastic counts were normalized by the volume of water sampled.

Microplastics that could be collected after visual microscopy were analyzed with ATR - FTIR using a Thermo Scientific Nicolet iS50 FT-IR Spectrometer to determine the polymer material and further confirm the melt test. The FTIR spectra were collected on a diamond crystal ATR cell with collection parameters including scan range 4000 - 400 cm^{-1}, 64 scans per sample with a resolution of 4 cm^{-1}. A background was collected prior to each particle analysis. Upon spectrum

collection, the built-in Hummel Polymer Library in the Omnic software was used as one tool to identify the polymer type, in addition to comparing the spectra to known standards.

FTIR Microscopy (μFTIR)
The Nicolet Continuum Infrared Microscope was used to analyze the > 45 μm and >106 μm size fractions. Aluminum oxide filters were scanned via a μFTIR building off previous research from Fox et al., 2022.[3] This work optimized the collection of these spectra by establishing collection settings of 32 scans per pixel at 4 cm^{-1} resolution using an IR aperture of 50 μm and a spectral range of 1300-4000 cm^{-1}. Transmission mode was also used in this study; transmission mode inherently produces higher-quality spectra than reflection. To achieve data collection in transmission mode, an Anodisc filter holder was constructed by making a 3D printed polylactic acid (PLA) microscope slide with an 18 mm diameter hole, larger than the 16 mm diameter of the center of an Anodisc, but smaller than the total 25 mm diameter of the Anodisc, which includes the polyethylene edge ring used to add structural integrity to the filter. Glass slides were placed on each side of the filter to secure the Anodisc in place. 2 mm by 2 mm grids were collected automatically, resulting in 1600 spectra per grid. 10 different grids, randomly spaced but not overlapping, were collected per sample resulting in 19.5 % percent of the Anodisc being examined. That data was extrapolated to estimate the particles on the full filter. This percentage represents a compromise, where analysis time and data size are manageable and count variance and fractional abundance of the major polymers appears acceptable; however, rare polymers may be under-or over-reported as determined in studies using similar μFTIR protocols.[5, 6] Recent work applying Raman spectroscopy to microplastic samples shows a similar conclusion, where 50% filter coverage is recommended but 20% coverage of sample boxes yields an average sampling error of ~20 ± 15%.[7]

Corrections applied to the spectra to make them more convenient to read included converting the % transmission to absorbance and applying an atmospheric suppression and baseline correction. To further matrix correct for natural-water samples, a background spectrum from the sample filter was identified (i.e., a spectrum with no microplastic particles) and subtracted from all the μFTIR spectra. The instrument then rendered a C-H sensitive heat map (based upon the 2850 cm^{-1} peak) allowing for the plastic particles to be more readily discovered and recorded. Within the spectral heat maps, each highlighted pixel corresponding to a unique region on the filter was investigated by looking at the full FTIR scan. The corrections aided in the visualization of the 2850 cm^{-1} C-H spectral peak, which is the paramount peak used here to define plastics, in addition to making the fingerprint region (1700-1300 cm^{-1}) easier to interpret for polymer identification. The pixels that exhibited the 2850 cm^{-1} plastic peak had their FTIR spectra analyzed using the built-in Hummel Polymer Library in OMNIC and compared to other reference spectra to determine particle type. A 50% match percentage was used to determine whether a particle was plastic and to establish the specific polymer variant. This process was repeated for each individual field of view (10 per sample). The counts per field of view were compiled and coupled with volume information to determine plastic particles per liter of sample water.

Flow Cytometry Analyses:
The 5-45 µm size fraction from the Cascade tower samples was oxidized and density-extracted as described above, then Nile red stained and sorted using a BD Influx Mariner (Becton Dickinson, formerly Cytopeia) at the Center for Aquatic Cytometry at Bigelow Laboratory for Ocean Sciences (East Boothbay, Maine, USA). A combusted salt (NaCl) solution (15 ppt) was used as sheath fluid. Microplastic detection was based on relative green fluorescence after excitation with a 488 nm laser-light and forward scatter (FSC) signatures for particles > 3 µm (see Minor et al., 2023 for further details of the flow cytometry of these lake samples, a field blank and positive control samples).[8]

QA/QC — Results of Sampling Blanks
A variety of ambient, field sampling, and processing blanks were run to ensure our counts were not biased by background signals from the sampling and processing.

Cascade Filter Sampling Blanks: The cascade filtration method was assessed by pumping 179.13 L of <0.8 um deionized water that had been placed in an HDPE barrel. This water was pumped from the barrel through the metal sieve stack using the diaphragm pump at 6.6 L/min. 10% of each barrel blank filter was scanned using the Nicolet Continuum Infrared Microscope.

For the >106 µm sieved barrel blank, 3 brown fragments were found. One was nylon, 1 was poly(ethylene:propylene) and the other was PE/PP. This represents a concentration of 0.17 MP/L. For the 45-106 um size range, 7 fragments were found. Five were white, 2 were brown and all were identified as PE. These counts represent a blank concentration of 0.39 MP/L. Based upon these low concentration values and the fact that these may include particles from the white HDPE barrel used to hold the blank water prior to pumping and not used in field sample work, we made no blank corrections to the cascade-collected field samples.

McLane Pump Field and Method Blanks: Two McLane pump blanks (Mc-A and Mc-B) were performed at 2 m depth at different locations in open water Lake Superior. Water was made "microplastic free" by placing a 5 µm filter above the 100 and 50 - µm filters. The presence of microplastics on the 100 and 50 µm filters allowed us to determine if the contamination originated from sample handling, i.e., pumping and subsequent sample processing. The McLane blanks underwent all sample processing steps (resuspension, oxidation, density extraction) and are thus full field sampling plus processing method blanks. Due to time constraints, 3.3% of each McLane blank filter was scanned using a Bruker Lumos II µFTIR.

For the McLane sampling and sample processing blanks, Mc-A >100 µm had 1 PP white fiber, and 1 polyoxymethylene (POM) translucent fragment; in Mc-A >50 µm, there were 7 polyoxymethylene (POM) fragments, 4 black and 3 translucent, 2 PP translucent fragments, and 1 polyamide translucent fiber. Thus the blank contribution to the lake estimated from Mc-A (summing both size fractions) would be 2.1 MP/L but this includes 1.4 MP/L from POM, which does not appear in any of our lake samples, and 0.2 MP/L from PA in the 45–100 um size range, while we find no PA in that size range in our lake samples. In Mc-B >100 µm, 1 translucent polyethylene terephthalate (PET) fiber, 1 white POM fragment, and 1 brown PP fragment were found; no plastics were found in the >50 µm fraction. Thus, the potential blank contribution is

1.0 MP/L but this includes POM, not found in any lake or harbor sample, and PET, which was only found in one size fraction of one lake sample (out of 16 total samples, 2 size fractions at 8 sites) and would be only 0.3 MP/L if the PP alone is considered. Based upon the relatively low contribution of particles per liter and the mismatch of polymer information between blanks and samples, no blank corrections to the McLane field data were performed.

Ambient blanks: Ambient blanks consisted of petri dishes open during sampling and sample processing. The ambient blank started with sampling Station C was resuspended by rinsing with MilliQ water and then filtering for visual microscopy with pressure and melt testing. Six microplastic particles were identified: 1 blue fiber, 2 clear fibers, 1 clear fragment, and 2 brown fragments. If the same number of microplastics were in the sample from station C, they would contribute 0.05 MP/L. The ambient blank started with Station K was resuspended by rinsing with MilliQ water and filtered onto an anodisc for analysis by uFTIR using the Nicolet Continuum instrument and scanning 10% of the filter. 6 plastic particles were identified in this blank: 2 poly(ethylene:propylene:diene), 2 nylon, 1 poly(butadiene) + naphthalene oil, and 1 polypropylene. If the same number of particles were in the sample for station K, they would contribute 0.22 MP/L.

QA/QC – Results of Positive Controls

Positive controls were used to evaluate recoveries in sampling, sample processing, and analyses.

Cascade Filter Recovery: Recovery from the cascade sieves was tested using visual microscopy and a standard consisting of PE spheres (600-710 µm, Cospheric, Product ID: CPB-0.96), PVC fragments (250 µm, bought from Goodfellow, manufactured by Ineos, product code CV316010), and PMMA spheres (85 µm, Goodfellow, 729-305-51), all used as obtained from the supplier and added to MilliQ water. We obtained 68% recovery by particle number for cascade filtration (sum of recovery on 300, 106 and 45 µm sieves). There was some breakthrough of larger particles into smaller sieves (out of 8 total treatments large particles found in >106 µm sample 3 times, and in >45 µm sample 1 time). Recovery from 300 µm Nylon filters (such as used in the McLane pumps) was tested in Fox et al 2022; 95.5% mass recovery of 600 µm PE spheres was determined and visual microscopy showed no visible degradation of the particles. Positive controls for the oxidation and density extraction steps were also published previously.[3] These varied by polymer type, with 93% recovery by particle count for MDPE (~350 µm size), 78% for PS (~250 µm size), 81 % for PET(~300 µm) 12% for PVC (~250 µm size), and 77% for PP (variable sizes).

µFTIR Recovery: The Thermo µFTIR positive control standard was directly filtered onto an Anodisc with 10% of the filter then counted. The standard was previously tested for microplastics concentrations using visual microscopy, and these were considered "true" values. This test was performed in triplicate (i.e., three separate filters were prepared). Recovery of PA 55 µm powder (Goodfellow, AM306055/1) from this step was 0–9.8% (mean 7%) and appeared to be at the lower size range of what we could identify with our µFTIR settings, which included a 50-µm pixel size. Recovery of PMMA 85 µm spheres (Goodfellow, 729-305-51) was (161–238%, mean 202%) and clumping of these was noticed on the filter. Recovery of MDPE (250 to 350 µm, Goodfellow, EV306010) was 20-64%; including poly(ethylene:propylene)

identifications along with PE increases recovery to 30 to 74% (mean 48%). Thus we appear to have ~48 to 202 % recovery in the μFTIR step for particles from 85 to 350 μm in size. Propagating across all the steps (filtering, oxidation, density extraction and μFTIR) for PE indicates a 30% recovery by particle count for the cascade filtration samples. If mass % recovery and a lack of visual particle degradation can be used to convert to % particle recovery for resuspension off nylon filters, the total recovery for sample processing and analyses for the McLane pump samples based upon PE is ~43%. Note that these recoveries are for non-weathered and often hydrophobic particles in MilliQ water without any surfactant and are ground-truthed by visual microscopy, which itself is subject to error. Natural water samples generally contain more weathered particles[9, 10] and surfactant materials[11, 12] within them and thus the recoveries of microplastics in such samples is likely to be higher.

Statistical Analysis of Station C:

Many samples needed to be split equally between more than one Anodisc to be optically thin enough for scanning via the Nicolet Continuum μFTIR. For Station C 106 μm, one of the two Anodiscs used was too optically thick due to clay content. Because resuspending the filtrate off the Anodisc could result in a significant loss of plastic particles, we estimated Station C plastic content from the one Anodisc only, multiplying the value by 2 for the final count reported. In an attempt to constrain the effect this might have on counts, the other three harbor stations 106 μm size fractions were split and filtered onto two Anodiscs, scanned and counted. The polymer counts were compared using F-tests and then T-tests between the two Anodiscs for each station using the Data Analysis ToolPak (Excel, Microsoft 365 MSO, version 2311). Station K, Station B and Station A had no statistical difference (95% confidence level) of polymer counts between Anodiscs when the sample was split in half. While the doubling of counts for Station C resulted in an elevated concentration compared to the other harbor stations, this value likely reflects Station C's proximity to major port facilities and the Duluth urban area.

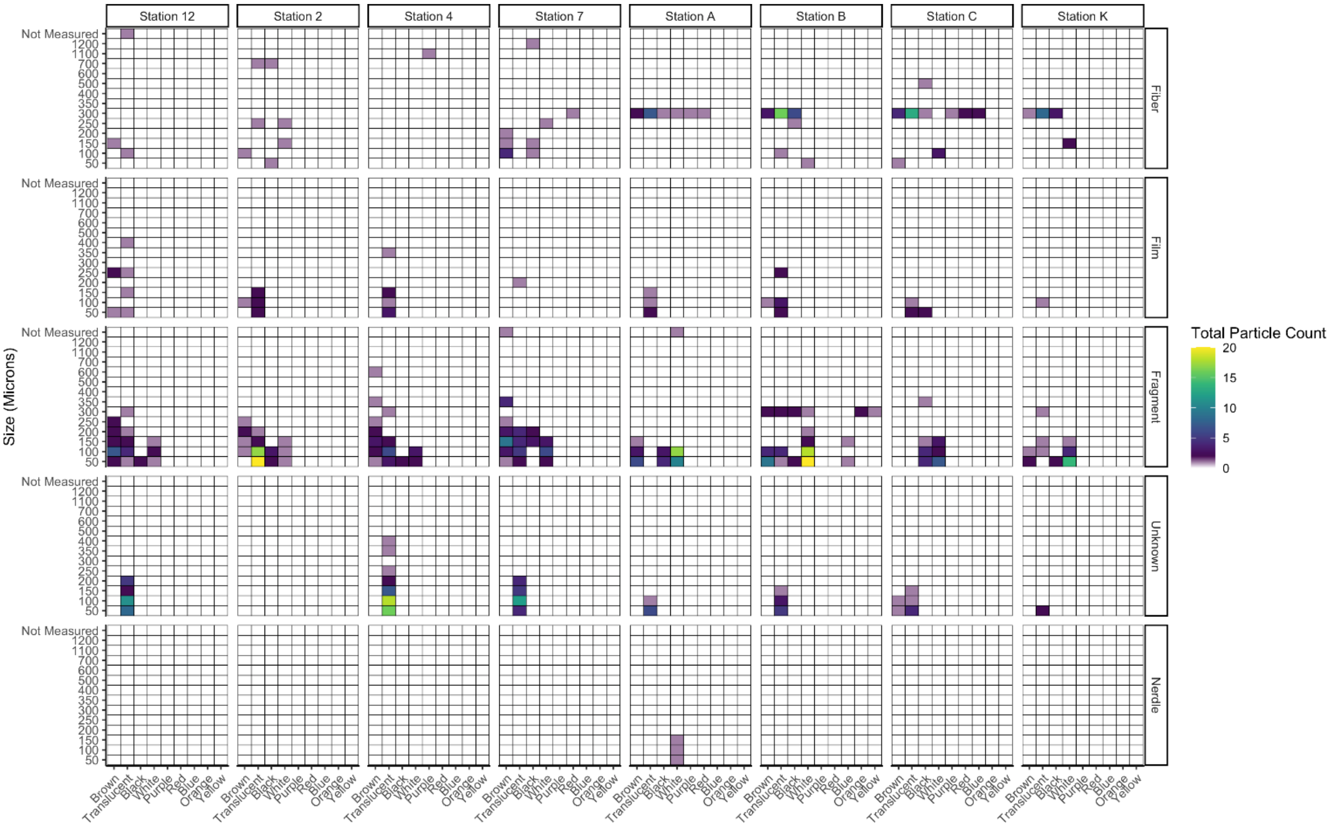

Figure S3. Color, morphology and size information for the cascade-sampled lake and harbor samples (1 m depth). Note that the y-axis (size) has a break in it between 700 and 1100 um in order to plot all the particles identified. Also note: harbor samples >300 um were not individually sized but listed as 300 um. Fibers and fiber clusters are considered the same morphology. Unknown generally indicates samples that did not extend beyond 1 pixel in uFTIR or because of clay content were hard to assess.

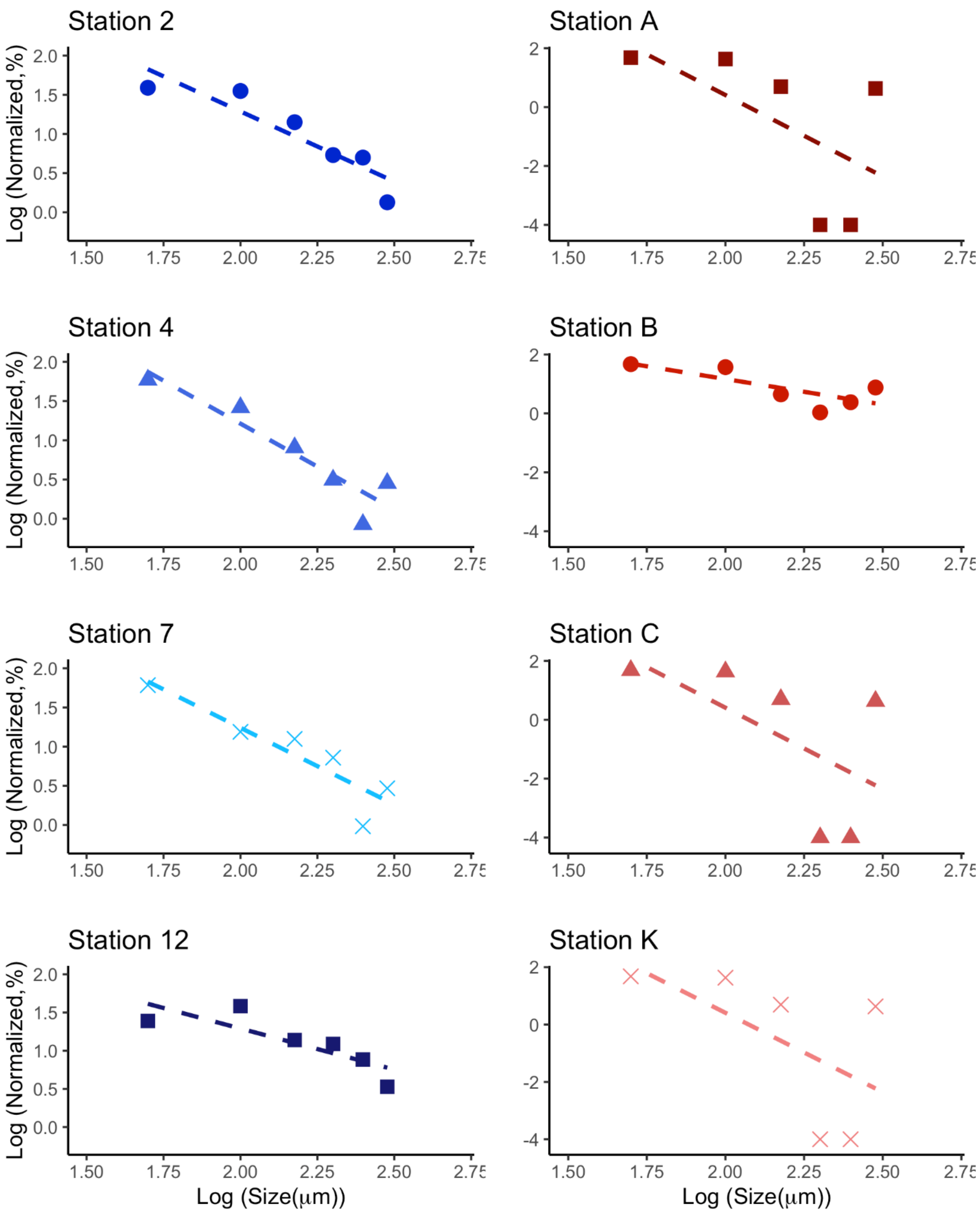

Figure S4. Size distribution power law plots for each individual station based upon averaged cascade and McLane pump data (thus 1 to 2 m depth) for the numbered (lake) stations and for cascade data (1 m depth) for the lettered (harbor) stations.

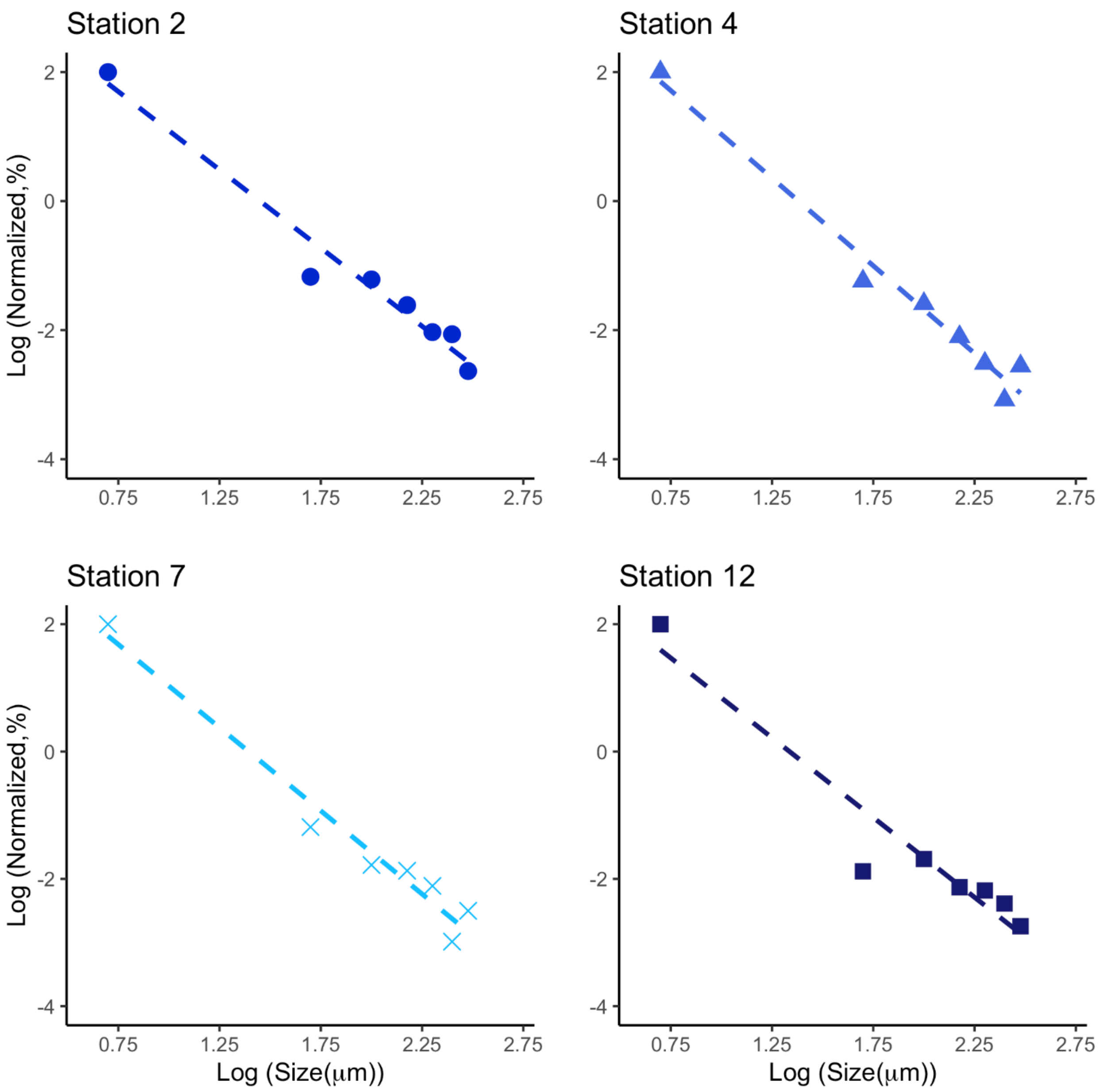

Figure S5. Power law data for the lake stations (both cascade and McLane pump data) with flow cytometry analyzed data from cascade sampling used for the smallest size fraction bin.

Table S1. Microplastic counts in each sieve fraction from cascade filtration and the total concentration per station.

**Harbor**	MP/L		**Lake**	MP/L
Station A			Station 2	
300 μm	0.049		300 μm	0.0088
106 μm	0.303		106 μm	0.591
45 μm	0.796		45 μm	0.832
Total Concentration	1.149		Total Concentration	1.432
Station B			Station 4	
300 μm	0.131		300 μm	0.00213
106 μm	0.960		106 μm	0.532
45 μm	0.634		45 μm	0.351
Total Concentration	1.725		Total Concentration	0.885
Station C			Station 7	
300 μm	0.197		300 μm	0.00442
106 μm	2.821		106 μm	0.177
45 μm	0.299		45 μm	0.796
Total Concentration	3.316		Total Concentration	0.978
Station K			Station 12	
300 μm	0.0479		300 μm	0.00255
106 μm	0.111		106 μm	0.395
45 μm	0.461		45 μm	0.433
Total Concentration	0.619		Total Concentration	0.831

Table S2. Microplastic counts in each McLane pump size fraction and the total concentration per station.

**Lake**	MP/L
Station 2	
50 µm	0.431
100 µm	0.295
300 µm	0.003
Total Concentration	0.729
Station 4	
50 µm	1.308
100 µm	0.313
300 µm	0.005
Total Concentration	1.626
Station 7	
50 µm	0.216
100 µm	1.089
300 µm	0.009
Total Concentration	1.314

Analysis of 50 µm standard particles:
In order to test the size limitations of the Nicolet Continuum µFTIR, a compound standard including a known number of plastic particles per mL in each size range was made. To represent the smallest size range Polyamide-Nylon 6(Goodfellow LS559036, AM306055/1) was used with a mean particle size of 55 microns. The standard was separated onto 3 aluminum oxide Anodiscs; 5 fields of view or about 10 % of the Anodisc was then analyzed via the Nicolet Continuum µFTIR. The resulting files were counted and recorded in the same manner as the samples in this study barring the statistical normalizer of the raw counts being multiplied by 10 instead of 5 to account for the area of the Anodisc not scanned. The average recovery rate of the Polyamide 6 was found to be 6.55%. Thus we assume that the 45 µm size fraction is being underrepresented in the counts of the samples of this study.

References

(1) Hendrickson, E.; Minor, E. C.; Schreiner, K. Microplastic Abundance and Composition in Western Lake Superior As Determined via Microscopy, Pyr-GC/MS, and FTIR. *Environmental Science & Technology* **2018**, *52* (4), 1787-1796. DOI: 10.1021/acs.est.7b05829.

(2) Minor, E. C.; Tennant, C. J.; Brown, E. T. A Seasonal to Interannual View of Inorganic and Organic Carbon and pH in Western Lake Superior. *Journal of Geophysical Research: Biogeosciences* **2019**, *124* (2), 405-419. DOI: https://doi.org/10.1029/2018JG004664 (acccessed 2024/04/02).

(3) Fox, J. M.; Schwoerer, G. D.; Schreiner, K. M.; Minor, E. C.; Maurer-Jones, M. A. Microplastics in the Water Column of Western Lake Superior. *ACS ES&T Water* **2022**, *2* (10), 1659-1666. DOI: 10.1021/acsestwater.2c00169.

(4) Lenaker, P. L.; Baldwin, A. K.; Corsi, S. R.; Mason, S. A.; Reneau, P. C.; Scott, J. W. Vertical Distribution of Microplastics in the Water Column and Surficial Sediment from the Milwaukee River Basin to Lake Michigan. *Environmental Science & Technology* **2019**, *53* (21), 12227-12237. DOI: 10.1021/acs.est.9b03850.

(5) Mintenig, S. M.; Kooi, M.; Erich, M. W.; Primpke, S.; Redondo- Hasselerharm, P. E.; Dekker, S. C.; Koelmans, A. A.; van Wezel, A. P. A systems approach to understand microplastic occurrence and variability in Dutch riverine surface waters. *Water Research* **2020**, *176*, 115723. DOI: https://doi.org/10.1016/j.watres.2020.115723.

(6) Löder, M. G. J.; Kuczera, M.; Mintenig, S.; Lorenz, C.; Gerdts, G. Focal plane array detector-based micro-Fourier-transform infrared imaging for the analysis of microplastics in environmental samples. *Environmental Chemistry* **2015**, *12* (5), 563-581. DOI: https://doi.org/10.1071/EN14205.

(7) Brandt, J.; Fischer, F.; Kanaki, E.; Enders, K.; Labrenz, M.; Fischer, D. Assessment of Subsampling Strategies in Microspectroscopy of Environmental Microplastic Samples. *Frontiers in Environmental Science* **2021**, *8*, Original Research. DOI: 10.3389/fenvs.2020.579676.

(8) Minor, E. C.; Gomes, U. D.; Schreiner, K. M.; Poulton, N. J.; Hendrickson, E.; Maurer-Jones, M. A. Small microplastic particles in Lake Superior: A preliminary study coupling Nile red staining, flow cytometry and pyrolysis gas chromatography–mass spectrometry. *Limnology and Oceanography: Methods* **2023**, *21* (12), 800-813. DOI: https://doi.org/10.1002/lom3.10582 (acccessed 2024/04/01).

(9) Arp, H. P. H.; Kühnel, D.; Rummel, C.; MacLeod, M.; Potthoff, A.; Reichelt, S.; Rojo-Nieto, E.; Schmitt-Jansen, M.; Sonnenberg, J.; Toorman, E.; Jahnke, A. Weathering Plastics as a Planetary Boundary Threat: Exposure, Fate, and Hazards. *Environmental Science & Technology* **2021**, *55* (11), 7246-7255. DOI: 10.1021/acs.est.1c01512.

(10) Andrady, A. L. Weathering and fragmentation of plastic debris in the ocean environment. *Marine Pollution Bulletin* **2022**, *180*, 113761. DOI: https://doi.org/10.1016/j.marpolbul.2022.113761.

(11) Klečka, G.; Persoon, C.; Currie, R. Chemicals of Emerging Concern in the Great Lakes Basin: An Analysis of Environmental Exposures. In *Reviews of Environmental Contamination and Toxicology Volume 207*, Whitacre, D. M. Ed.; Springer New York, 2010; pp 1-93.

(12) Das, R.; Hoysall, C.; Rao, L. Unveiling the origin, fate, and remedial approaches for surfactants in sewage-fed foaming urban (Bellandur) Lake. *Environmental Pollution* **2023**, *339*, 122773. DOI: https://doi.org/10.1016/j.envpol.2023.122773.
